# Metabolic Adaptation and Pulmonary ceRNA Network Plasticity in *Orientallactaga sibirica* During Water Deprivation Stress

**DOI:** 10.3390/ijms27031458

**Published:** 2026-02-01

**Authors:** Yongling Jin, Rong Zhang, Xin Li, Linlin Li, Dong Zhang, Yu Ling, Shuai Yuan, Xueying Zhang, Heping Fu, Xiaodong Wu

**Affiliations:** 1 College of Grassland Science, Inner Mongolia Agricultural University, Hohhot 010020, China; jinyongling2019@163.com (Y.J.); zhangrong20212021@126.com (R.Z.); lixin9623@163.com (X.L.); lilinlin2023007@163.com (L.L.); zhangdong@imau.edu.cn (D.Z.); lingyuolcuc@126.com (Y.L.); yuanshuai2020@163.com (S.Y.); fuheping@126.com (H.F.); 2 State Key Laboratory of Animal Biodiversity Conservation and Integrated Pest Management, Institute of Zoology, Chinese Academy of Sciences, Beijing 100101, China; zhangxy@ioz.ac.cn

**Keywords:** desert rodents, water deprivation stress, lung, full-length transcriptome sequencing, ceRNA

## Abstract

Rising global temperatures lead to a continuous increase in the frequency and intensity of extreme weather events, such as droughts and floods, posing serious threats to terrestrial homeotherms. However, adaptive changes in respiratory metabolism and molecular mechanisms in lung tissues of small mammals under extreme water shortage conditions remain unclear. This study hypothesized that small desert mammals can adapt to extreme water shortage environments by regulating the plasticity of lung tissue gene expression and respiratory metabolism. Using 29 wild-caught Siberian jerboas (*Orientallactaga sibirica*) as subjects, we implemented a 12-day complete water deprivation protocol to simulate extreme aridity. Body weight, food intake, and daily energy expenditure (DEE) were monitored throughout the experiment. Whole-transcriptome sequencing of lung tissues was performed to profile mRNA, circRNA, and miRNA expression, with competitive endogenous RNA (ceRNA) network analysis to explore molecular mechanisms underlying lung adaptation to water deprivation. Over the 12-day water deprivation (WS) period, *Orientallactaga sibirica (O. sibirica)* exhibited a 30.3% reduction in body mass and a 68.1% decrease in food intake relative to the baseline level. DEE during the peak activity period at the end of the experiment was 12.6% lower in the WS group compared to the control group. In lung tissue, structural integrity-related genes (*Mybl2*, *Ccnb1*) were downregulated. A key finding was that *circ_0015576* exhibits a significant positive correlation with the potassium channel gene *Kcnk15* and a robust negative correlation with *miR-503-5p*—suggesting that *circ_0015576* functions as a competing endogenous RNA (ceRNA) to sequester *miR-503-5p* and thereby derepress *Kcnk15* expression. Core regulatory genes (*ApoA4*, *Dusp15* etc.) were also coordinately downregulated. Collectively, these results indicate that *O. sibirica* reduces overall energy expenditure, which may be associated with lung gene expression plasticity, such as those related with lung cell proliferation, pulmonary function, and gas exchange efficiency. This metabolic downregulation facilitates energy conservation under severe water scarcity.

## 1. Introduction

Climate change intensifies extreme events like droughts globally, as noted in the IPCC Sixth Assessment Report [1,2,3,4]. Deserts—characterized by extreme aridity, dramatic temperature fluctuations [5,6,7,8], and intense ultraviolet radiation—pose core challenges to homeotherms for maintaining body temperature and conserving water [9,10,11,12]. To cope with these challenges, desert organisms have evolved distinct adaptive traits [13,14,15,16,17], such as a low basal metabolic rate (BMR), low evaporative water loss (EWL), and large relative medullary thickness (RMT) [18,19,20,21,22,23,24]. Genomic and metabolomic convergent adaptations (e.g., fat metabolism regulation, renal Aquaporin family overexpression) further support their survival under arid stress [25,26,27].

Animal drought adaptation research has predominantly focused on the kidney, a pivotal organ orchestrating water conservation and retention. Extensive investigations into urine concentration and water–salt reabsorption processes have elucidated a comprehensive theoretical framework, encompassing the activation of osmoregulatory signaling cascades, fine-tuned regulation of key membrane transporters (e.g., aquaporin (*Aqp*) family) [26,28,29,30,31], and species-specific amino acid substitutions in functionally critical genes such as uromodulin (*Umod*) and sodium channel, nonvoltage—gated 1 alpha subunit (*Scnn1a*) [25,32]. Nevertheless, these studies remain narrowly confined to a kidney-centric paradigm, largely neglecting the synergistic contributions of other major organs (e.g., lungs) to the maintenance of drought resilience in terrestrial homeotherms. As a representative desert rodent, *O. sibirica* has evolved specialized water-saving strategies, including enhanced metabolic water production and adaptive osmoregulatory modifications [25], which underscores the imperative of investigating understudied organ systems to fully decipher the multi-organ coordinated mechanisms underlying drought adaptation.

Water deprivation, a hallmark of arid environments, directly elicits oxidative stress and perturbs ionic homeostasis in the lungs of desert rodents [33,34,35]. This cascade of stress responses activates inflammatory signaling pathways (e.g., nuclear factor-κβ (NF-κβ)), prompting the release of pro-inflammatory cytokines such as interleukin-6 (Il-6) and tumor necrosis factor-α (Tnf-α), which in turn induce pulmonary inflammatory injury and disrupt tissue integrity [33,36,37]. Persistent drought-induced oxidative stress and inflammation further trigger apoptosis of alveolar epithelial and endothelial cells via mitochondrial (e.g., cytochrome C release) and death receptor (e.g., Fas/FasL) pathways [38,39,40,41,42] Concurrently, water–electrolyte imbalances (e.g., hypernatremia, hypokalemia) resulting from water scarcity impair energy metabolism in lung cells and inhibit the activity of key transport proteins like Na^+^/K^+^-ATPase, leading to intracellular ion disequilibrium and functional dysfunction [43,44,45]. In summary, these pulmonary adaptive responses to water deprivation compromise respiratory efficiency and alter energy expenditure; yet the molecular regulatory networks governing how the lungs of *O. sibirica* cope with water deprivation stress remain poorly elucidated.

Circular RNAs (circRNAs) are evolutionarily conserved non-coding RNAs produced by back-splicing, and accumulating evidence robustly supports their core regulatory function as competitive endogenous RNAs (ceRNAs) that sequester microRNAs (miRNAs) [46,47,48,49,50,51]. MicroRNAs (miRNAs) are short non-coding RNAs that post-transcriptionally regulate target mRNAs by inhibiting translation or inducing degradation, thereby serving as key modulators of diverse biological pathways [52,53]. Notably, under environmental stress, circRNA expression can be dynamically regulated through the binding of regulatory factors to intronic sequences, enabling them to modulate downstream physiological processes via miRNA sponging [51,54,55]. However, most investigations into circRNA–miRNA–mRNA regulatory networks have focused on disease models, with relatively few exploring their implications in animal adaptive mechanisms, especially in the context of stress responses [56,57,58,59,60,61,62]. Furthermore, traditional research on animal adaptation has relied heavily on model organisms such as C57BL/6J (*Mus musculus*) under controlled laboratory conditions [28], which neglects the complex real-world stressors (e.g., diurnal temperature fluctuations, food resource variability, predation pressure) encountered by wild rodents [63,64].

To investigate whether the lungs of the desert-adapted *O. sibirica* exhibit unique adaptive mechanisms under water deprivation stress, we used wild-caught individuals placed in an indoor semi-natural environment. Artificial water-deprivation stress (WS group) simulated extreme drought conditions. Differentially expressed circRNAs, miRNAs, and mRNAs in *O. sibirica* lungs were identified. Combined with enrichment analysis, we predicted the biological functions of differentially expressed mRNAs and the potential signaling pathways. By constructing a circRNA–miRNA–mRNA regulatory network, we further explored the potential role of non-coding RNA competition in the water stress adaptation mechanisms of differentially expressed mRNAs.

## 2. Results

### 2.1. Alterations in Physiological Traits of O. sibirica After Water Deprivation Stress

**Figure 1:**
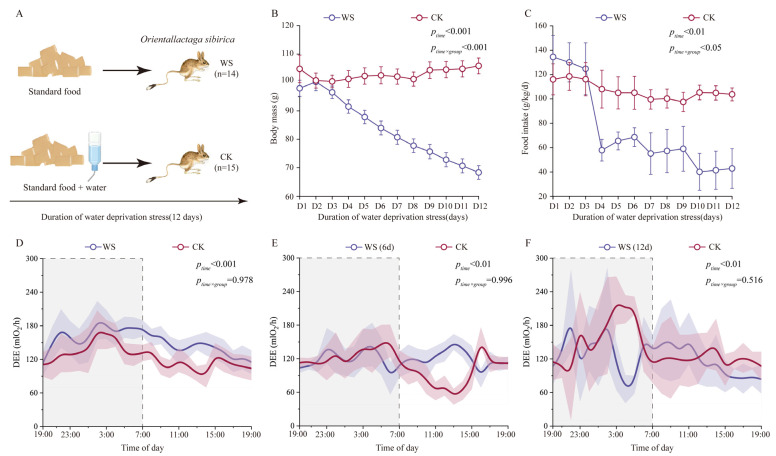
Effects of water deprivation stress on body mass, food intake, daily energy expenditure, and blood parameters in *O. sibirica*. (**A**) Schematic representation of the experimental design. (**B**) Body mass dynamics during the stress period. (**C**) Food intake. (**D**–**F**) Daily energy expenditure (DEE) was measured before water deprivation stress (**D**), after 6 days (**E**), and after 12 days (**F**) of water deprivation stress; shaded regions indicate periods of high locomotor activity (19:00–7:00). WS, water deprivation stress; CK, control conditions. The shaded grey areas indicate periods of high locomotor activity.

During the 12-day treatment period (Figure 1A), the body mass of *O. sibirica* in the water deprivation stress group (WS) was significantly lower than that under control conditions (CK) (*F*_11,165_ = 31.804, *p* < 0.001), and a significant interaction was observed between group and acclimation time (*F*_11,165_ = 48.411, *p* < 0.001) (Figure 1B). Concurrently, water deprivation stress led to a significant stage-wise decrease in food intake (*F*_11,165_ = 9.789, *p* < 0.01), with a significant interaction between water deprivation stress and stress duration (*F*_11,165_ = 5.036, *p* < 0.05) (Figure 1C). The body mass change over time showed a difference between the two groups. The control group remained stable throughout the process, whereas the body mass and food intake of the stress group decreased by 30.3% and 68.1% relative to the initial levels, respectively. The sustained weight loss and reduced food intake in *O. sibirica* under water deprivation stress were in line with the features of a negative energy balance.

The daily energy expenditure (DEE) of *O. sibirica* was generally higher at night than during the day, exhibiting a significant time effect (*F*_23,230_ = 3.091, *p* < 0.001); however, the interaction effect between time and grouping was not significant (*F*_23,230_ = 0.489, *p* = 0.978) (Figure 1D). After 6 days of stress, the DEE of *O. sibirica* (with body mass as a covariate) was not affected during the peak activity period (*F*_1,141_ = 0.086, *p* = 0.770) but was significantly higher than the control group during the non-peak activity period from 12:00 to 15:00 (*F*_1,141_ = 7.572, *p* < 0.05) (Figure 1E). After the 12-day stress period, when normalized to body mass, the DEE of the stress group jerboas was 12.6% lower compared to the control group at the onset of the peak activity period (*F*_1,141_ = 22.806, *p* < 0.05) (Figure 1F).

### 2.2. Differentially Expressed Coding and Non-Coding RNAs Induced by Water Deprivation Stress

**Figure 2:**
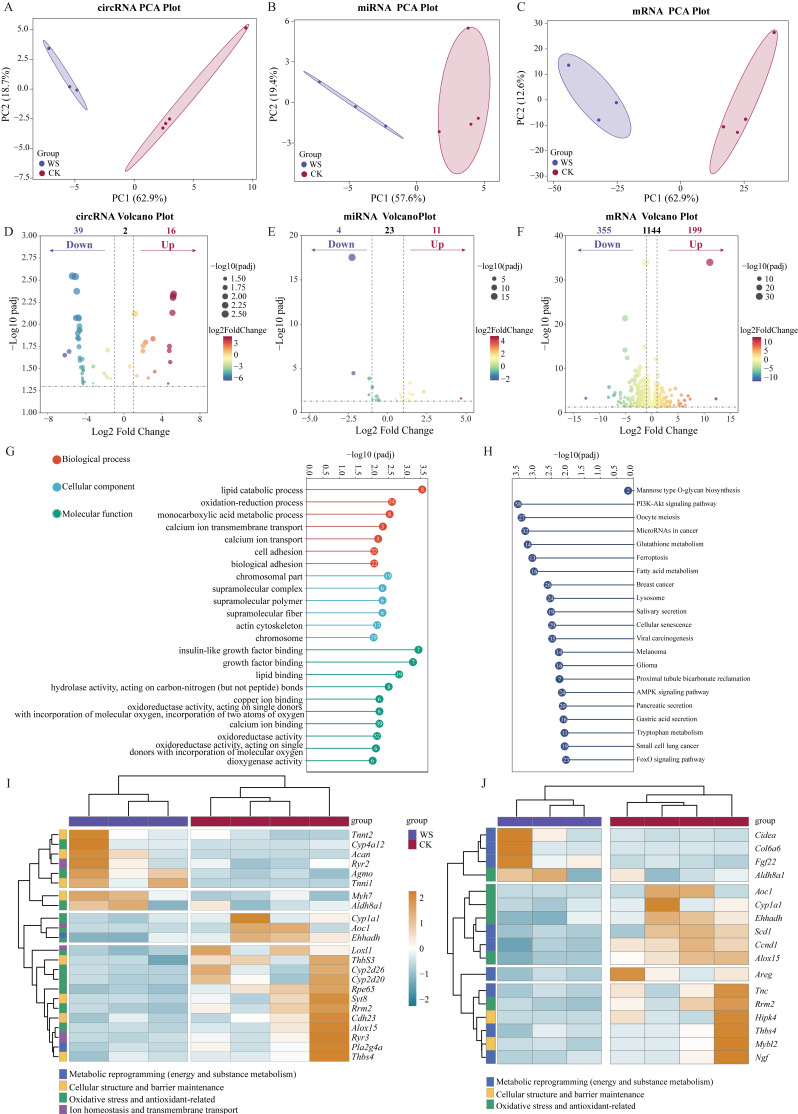
Differential gene expression and functional enrichment analysis of lung tissues in water deprivation stress (WS) versus control (CK) groups. (**A**–**C**) Principal component analysis (PCA) of differentially expressed RNAs. The shadow area represents the confidence interval of the sample. (**D**–**F**) Volcano plots of differentially expressed RNAs. (**G**) GO functional enrichment analysis of differentially expressed mRNAs. (**H**) KEGG pathway enrichment analysis of differentially expressed mRNAs. (**I**) Heatmap of core differentially expressed genes from GO functional categories. (**J**) Heatmap of core differentially expressed genes from KEGG pathways.

Principal component analysis (PCA) revealed a clear separation of circRNA, mRNA, and miRNA expression profiles between the water deprivation stress group (WS, *n* = 14, pooled into three biological replicates) and control group (CK, *n* = 15, pooled into four biological replicates) in lung tissues. For circRNAs, PC1 explained 62.9% of the total variance and PC2 18.7% (cumulative 81.6%), supporting distinct clustering (Figure 2A). For miRNAs, PC1 (57.6%) and PC2 (19.4%) accounted for 77.0% of the total variance, with obvious group separation (Figure 2B). For mRNAs, PC1 (62.9%) and PC2 (12.6%) contributed 75.5% of the cumulative variance, confirming group separation (Figure 2C). These results validate significant differences in coding and non-coding RNA expression patterns between groups, laying a reliable foundation for subsequent differential expression analysis. At the circRNA level, a total of 55 differentially expressed transcripts were identified (16 upregulated and 39 downregulated); at the miRNA level, 15 were identified (11 upregulated and 4 downregulated); and at the mRNA level, 554 were detected (199 upregulated and 355 downregulated) (Figure 2D–F). Hierarchical clustering analysis based on expression levels of the obtained differentially expressed transcripts revealed clear partitioning between WS and CK groups for different RNA types, with good consistency among replicates (Supplementary Figure S1A–C).

GO enrichment analysis of differentially expressed mRNAs showed that these genes were primarily enriched in four key biological processes: energy and substance metabolism, oxidative stress and antioxidant defense, ion balance and transmembrane transport, and cell structure and barrier maintenance (padj < 0.05, Figure 2G; Supplementary Table S1). To identify the core genes functionally linked to drought tolerance, we further screened genes from these four processes based on their annotated functions in drought adaptation. In terms of energy and substance metabolism-related functions, differentially expressed mRNAs were significantly enriched in lipid catabolic process (GO:0016042) and monocarboxylic acid metabolic process (GO:0032787). Core genes included the fatty acid β-oxidation-related gene enoyl-CoA hydratase, and 3-hydroxyacyl-CoA dehydrogenase (*Ehhadh*) was significantly downregulated (Figure 2I,J), while the key lipid metabolism gene alkylglycerol monooxygenase (*Agmo*) and the aldehyde metabolism gene aldehyde dehydrogenase 8 family member a1 (*Aldh8a1*) were significantly upregulated. In the oxidative stress and antioxidant defense pathway, DEGs were significantly enriched in oxidation-reduction process (GO:0055114), oxidoreductase activity (acting on single donors with incorporation of molecular oxygen, incorporation of two atoms of oxygen; GO:0016702), and oxidoreductase activity (GO:0016491). Core genes included several members of the *Cyp* family (*Cyp1a1*, *Cyp2d20*, etc.), which were downregulated. Regarding ion balance and transmembrane transport-related functions, DEGs were significantly enriched in copper ion binding (GO:0005507) and calcium ion transport (GO:0006816), with the core gene being the calcium ion transport gene ryanodine receptor 3 (*Ryr3*), which was significantly downregulated. For cell structure and barrier maintenance-related functions, DEGs were significantly enriched in actin cytoskeleton (GO:0015629), cell adhesion (GO:0007155), and biological adhesion (GO:0022610). Core genes included the extracellular matrix synthesis gene aggrecan (*Acan*) and muscle fiber structure genes myosin heavy chain 7 (*Myh7*), troponin I type 1 (*Tnni1*), and troponin T type 2 (*Tnnt2*), which were significantly upregulated (Figure 2G,I).

KEGG pathway enrichment analysis showed that DEGs were significantly enriched in pathways such as the PI3K-Akt signaling pathway (rno04151), glutathione metabolism (rno00480), ferroptosis (rno04216), and fatty acid metabolism (rno01212) (padj < 0.05, Figure 2H; Supplementary Table S2). From these pathways, there were 18 key genes (e.g., *Aldh8a1*, arachidonate 15-lipoxygenase (*Alox15*), etc.), among which *Aldh8a1*, *Alox15*, and five others overlapped with GO core genes, which are involved in pathways such as lipid metabolism (*Ehhadh*, stearoyl-CoA desaturase 1 (*Scd1*)), oxidative stress (*Alox15*, amine oxidase, copper containing 1 (*Aoc1*)), and cellular stress (myb proto-oncogene like 2 (*Mybl2*), cyclin B1 (*Ccnb1*)) (Figure 2H,J).

### 2.3. CircRNA–miRNA–mRNA Regulatory Network and Functional Modules

**Figure 3:**
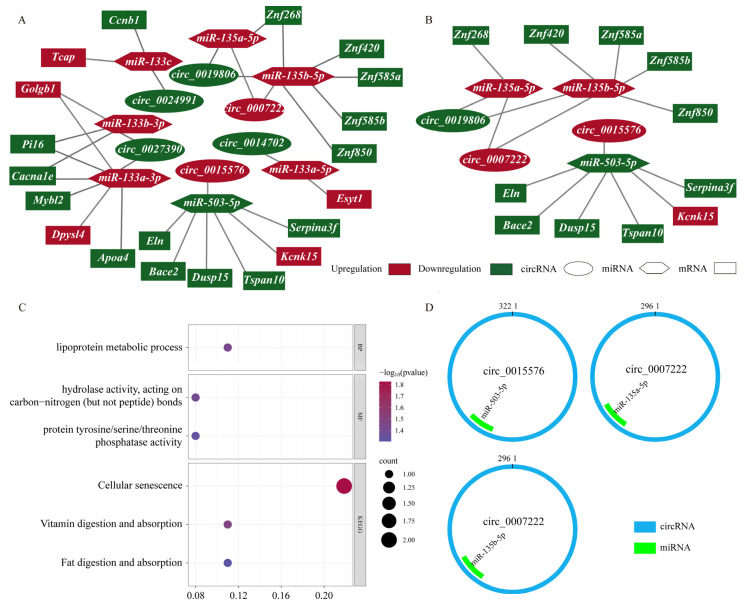
Comprehensive analysis of the circRNA–miRNA–mRNA regulatory network and identification of functional modules in lung tissue under water deprivation stress. (**A**) The circRNA–miRNA–mRNA regulatory network associated with the response to water deprivation stress. (**B**) The ceRNA regulatory network associated with the response to water deprivation stress. (**C**) The enrichment terms of differentially expressed mRNAs of the circRNA–miRNA–mRNA network in GO and KEGG analyses. (**D**) The predicted differential miRNA binding sites of circRNA in the circRNA–miRNA–mRNA regulatory network. circRNA is represented by the outer green circle, and miRNAs are represented by the inner red rectangle. The relative locations of miRNAs to circRNA represented the binding sites.

In this study, predicted miRNAs for the circRNA–miRNA pairs were further filtered by matching with the previously selected differentially expressed miRNAs, yielding information on differentially expressed circRNA–miRNA pairs (Supplementary Figure S2A; Supplementary Table S3). Target mRNAs of differentially expressed miRNAs were retrieved from miRanda and RNAhybrid. By matching with the previously selected differentially expressed mRNAs, predicted mRNAs for miRNA–mRNA pairs were further screened, yielding information on differentially expressed miRNA–mRNA pairs (Supplementary Figure S2B; Supplementary Table S3). Using circRNAs as baits, miRNAs as cores, and mRNAs as targets, a circRNA–miRNA–mRNA regulatory network was constructed (Figure 3A). This network contained six differentially expressed circRNAs (two upregulated, four downregulated), seven differentially expressed miRNAs (six upregulated, one downregulated), and twenty differentially expressed mRNAs (five upregulated, fifteen downregulated), comprising a total of 33 interaction pairs. Regarding the criteria for ceRNA network construction—namely, a positive correlation between circRNA and mRNA (r > 0.5) and a negative correlation between circRNA and miRNA (r < −0.5)—we further filtered significant correlation pairs (padj < 0.05) according to these conditions, and the resulting pairs were ultimately used to construct the ceRNA network. The ceRNA network was constructed using two circRNAs (one upregulated and one downregulated) as the capture agents, two upregulated and one downregulated miRNAs as the core, and three downregulated (*Apoa4*, *Cacnale,* and *Mybl2*) and one upregulated (*Kcnk15*) mRNAs as the targets (Supplementary Table S3; Figure 3B).

To further understand the functions of core mRNAs in the ceRNA network, GO enrichment and KEGG pathway analyses were performed. Pathway enrichment and functional annotation results showed significant enrichment in three key pathways: cellular senescence (rno04218), vitamin digestion and absorption (rno04977), and fat digestion and absorption (rno04975). Specific enrichment was also observed in functional terms, such as the lipoprotein metabolic process (GO:0042157), hydrolase activity, acting on carbon-nitrogen (but not peptide) bonds (GO:0016810), and protein tyrosine/serine/threonine phosphatase activity (GO:0008138) (Figure 3C).

The interaction between circular RNAs and miRNAs can be determined by the total predicted score of the predicted stable structure complex (the higher the value, the stronger the binding), as well as the free energy (the lower the value, the more stable the binding), but these are not conclusive. The combined analysis of the two indicators (the total score and total energy value) suggests a potentially stable interaction between *miR-503-5p* and *circ_0015576*, supporting the possibility of a functional binding relationship. However, these computational predictions should be interpreted with caution, as they remain indicative rather than definitive evidence (Figure 3D, Table 1).

### 2.4. RT-qPCR Validation of Key DEGs

To validate the sequencing and bioinformatics findings, we performed RT-qPCR on key RNAs identified in the circRNA-miRNA-mRNA network, with three biological replicates in each group, plus three technical replicates per sample. *GAPDH*, a stably expressed housekeeping gene, was used as the reference gene for normalization.

**Figure 4:**
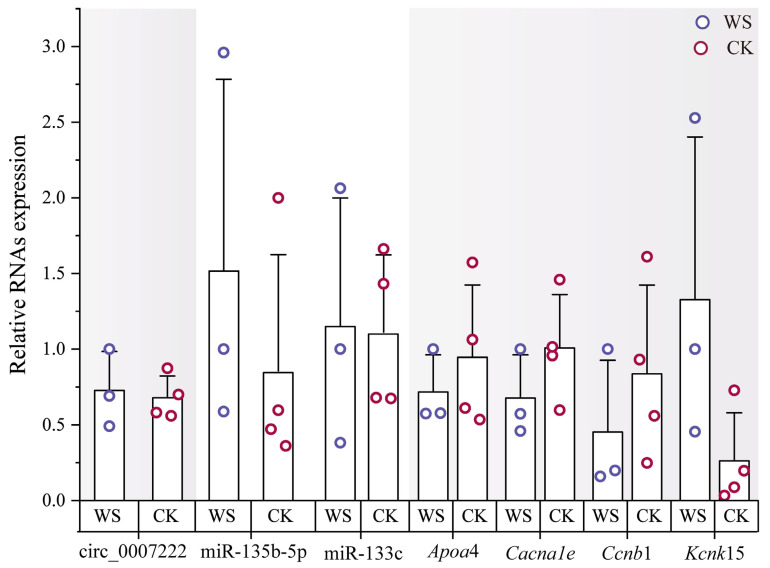
Expression of key differentially expressed RNAs measured through RT-qPCR. The shaded areas in the figure represent different types of RNAs. Note: Statistical significance (*p* < 0.05) is marked with an asterisk (*) for groups with significant differences, and no mark is used for groups without significant differences.

To validate the sequencing and bioinformatics findings, we performed RT-qPCR on key differentially expressed RNAs identified in the circRNA–miRNA–mRNA network. The expression patterns of circRNAs and miRNAs detected through qPCR closely matched those obtained through sequencing (Figure 4). Among the core RNAs, several associated with energy and substrate metabolism, including apolipoprotein A-IV (*ApoA4*), showed a tendency to decrease (*p* = 0.491). Likewise, genes related to oxidative stress and antioxidant defense, as well as ion homeostasis and transmembrane transport, showed corresponding changes. Calcium voltage-gated channel subunit alpha1 E (*Cacna1e*) showed a tendency to decrease (*p* = 0.245), while potassium two-pore domain channel subfamily K member 15 (*Kcnk15*) showed a tendency to increase (*p* = 0.111). Moreover, genes involved in cell structure and tissue homeostasis maintenance, such as *Ccnb1*, also showed a tendency to decrease (*p* = 0.397) (Figure 4). However, statistical analysis indicated that these expression changes did not reach significant levels. These qPCR results support the consistency of expression trends between sequencing and validation experiments, thereby corroborating the reliability of the sequencing and bioinformatic predictions for the circRNA–miRNA–mRNA network.

## 3. Discussion

The main challenges for species in extremely arid habitats are maintaining body temperature and conserving water [65]. Hypothermia can occur under cold arid conditions or nocturnal/winter cold in hot environments [65], while reduced adaptive thermogenesis [13,27,66,67] and pulmonary evaporative water loss are key water-conservation mechanisms in small rodents [68]. Shifting from lipid to carbohydrate metabolism increases metabolic water yield per oxygen molecule, aiding survival in water-limited settings. This study compared the metabolic characteristics and plasticity of the non-coding RNA regulatory network in the lungs of *O. sibirica* under free-drinking versus water-deprived conditions.

### 3.1. Energy Metabolism and Weight Regulation

Water deprivation stress induces species-specific responses in body mass, food intake, and energy metabolism, which depend on intrinsic drought adaptation backgrounds [28,69,70]. Desert-adapted species typically maintain core phenotypic stability through precise physiological regulation [28,71], whereas non-tolerant species exhibit marked phenotypic disruption [71,72,73]. *O. sibirica* shares a “stability maintenance tendency” with *Notomys alexis*, but the response magnitude shows species specificity [71]. A firmly supported finding is the significant but gradual body mass loss (approximately 30.3% decrease from baseline) in water-stressed (WS) animals, consistent with the “slow tolerance type” observed in desert-adapted species—contrasting with the steeper daily decline of 3.2–4.5% in non-desert *Rattus flavipectus* [74]. The food intake showed a significant stage-wise reduction (approximately 68.1% decrease from baseline), with intake per kilogram of body mass decreasing as the body mass declined, reflecting the species’ desert-specific capacity for buffering weight regulation. Notably, *O. sibirica*’s body mass continued to decline rather than stabilize despite reduced food intake—differing from *N. alexis* [71]. This may suggest a prioritization of internal energy reserve (e.g., fat) utilization to sustain basal metabolism during water deprivation, where reserve depletion outpaces the mass-sparing effects of reduced feeding. This pattern aligns with the “active energy reserve consumption” strategy reported in *N. alexis*, which relies on fat oxidation for metabolic water production [71]. Consistent with other desert rodents (e.g., *Gerbillus pusillus*, *N. alexis*, *Acomys russatuss*) [71,75,76], *O. sibirica* actively suppressed food intake to minimize water loss—a well-documented drought-adaptation mechanism. The DEE was significantly higher during the non-peak activity period at day 6 and only became significantly lower by 12.6% during the peak activity period at day 12 of WS. This trajectory parallels the reduced average daily metabolic rate (ADMR) observed in *G. pusillus* [76], suggesting that *O. sibirica* initiates metabolic suppression under prolonged WS stress, especially during high-energy-consumption periods, to enhance water conservation. However, cross-species comparisons of DEE patterns should be interpreted cautiously: similar phenotypic responses may arise from distinct underlying mechanisms or be influenced by experimental variables (e.g., duration, temperature, diet), not solely intrinsic adaptation.

### 3.2. Cell Cycle and Pulmonary Structural Remodeling

Given its dual role in energy metabolism and gas exchange, the lung may contribute to this dynamic metabolic regulation through functional adaptations (e.g., respiratory efficiency modulation, thermoregulatory breathing behaviors) [72].

**Figure 5:**
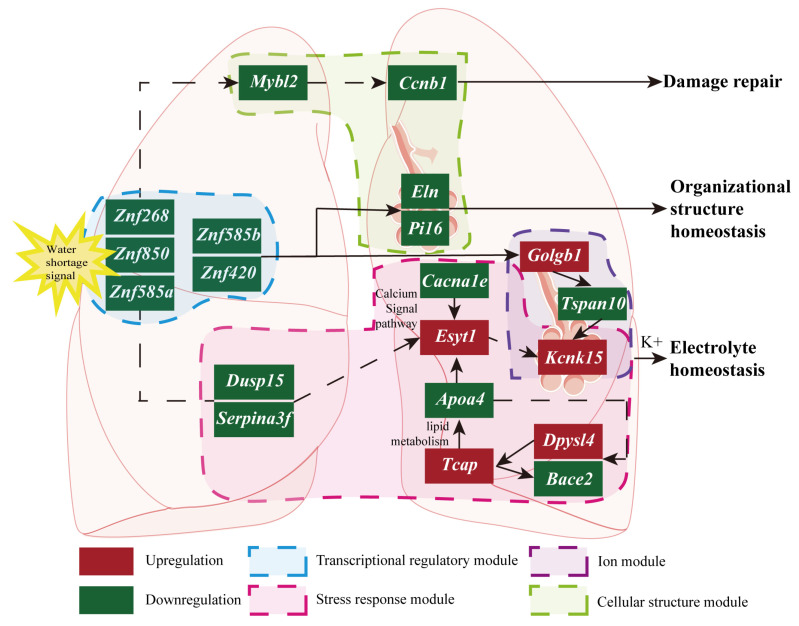
Interactions among pulmonary genes following drought stress in *O. sibirica*. Red denotes upregulated genes, and green denotes downregulated genes. Solid lines indicate direct interactions, while dashed lines indicate indirect interactions.

Network analysis of non-coding RNAs in the lung tissue of *O. sibirica* (Figure 5) exposed to WS indicated significant downregulation of the zinc finger protein family (*Znf268*, *Znf850*, *Znf585a*, *Znf585b*, *Znf420*). Znf family downregulation may affect downstream cell cycle regulators *Mybl2*, which is an important cell cycle regulator that promotes the transition from the G2 to the M phase [77], and *Ccnb1*, which is a key driver protein in this process [78]. Inhibition of this regulatory axis likely hinders the proliferation of important repair cells such as alveolar type II cells or fibroblasts, thereby impeding the recovery of the epithelial barrier and tissue structure [79,80]. However, persistent abnormal activation can lead to excessive cell proliferation and imbalance in tissue remodeling, which is the basis for the progression of many lung diseases (e.g., pulmonary fibrosis) [81,82]. Furthermore, a decrease in elastin (*Eln*) can directly lead to reduced lung tissue elasticity [83], while decreased Peptidase inhibitor 16 (*Pi16*) expression could alter fibroblast activation [84].

Both *Znf585b* and *Znf420* expressions were downregulated. Their binding capacity to the negative regulatory regions of the *Mybl2* and *Ccnb1* promoters weakened, relieving direct inhibition. However, the expression levels of *Ccnb1* and *Mybl2* still showed a downregulated trend, suggesting the dominant role of other regulatory mechanisms. Concurrently, the downregulation of *Znf268* expression attenuated its transcriptional activation of *Pi16*, resulting in reduced PI36 expression. This, in turn, diminished the repression of pro-inflammatory cytokines, including IL-6 and TNF-α, thereby compromising cellular anti-aging capacity [85,86,87,88]. Notably, dual-specificity phosphatase 15 (*Dusp15*) expression was downregulated, weakening its negative regulatory effect on the MAPK pathway. This theoretically leads to enhanced MAPK pathway activity (increased p-ERK levels), which would promote the transcriptional activation of *Ccnb1* and *Mybl2*. However, their actual expression remained downregulated, further confirming the complex checks and balances within multiple regulatory pathways [89,90]. This regulatory network differs from previous findings in mouse models: in mice, upregulation of *miR-133a-3p/133c* can directly reduce *Mybl2* and *Ccnb1* expression, decreasing the accumulation of senescence markers p53 and p21 [91,92]. However, *O. sibirica* may maintain anti-aging network function via synergistic transcriptional and post-transcriptional balance—even with Znf factor downregulation. This working model aligns with broader observations that Znf family genes regulate cell proliferation, apoptosis, and epithelial–mesenchymal transition (EMT) in various diseases [93]. According to functional annotations derived from the literature, the mechanisms underlying cell cycle regulation and its associated transcriptional control, as well as lung structural adaptation, remain hypothetical and require experimental validation.

### 3.3. Candidate Regulatory Network of ceRNA

This study showed that multiple interrelated pathways may be associated with the upregulation of *Kcnk15* expression. *Kcnk15* encodes the two-pore potassium channel (K2P15.1), a key channel protein for maintaining resting membrane potential and potassium ion balance [94]. Our results suggest that at least two independent signaling axes may be involved in regulating *Kcnk15*. One involves downregulation of the zinc finger protein family acting through promoting golgin b1 (*Golgb1*) and inhibiting tetraspanin 10 (*Tspan10*) [95,96,97,98,99,100,101]. The other axis involves zinc finger protein downregulation indirectly inhibiting the *Dusp15* and serine protease inhibitor a3f (*Serpina3f*) complex. *Dusp15* participates in cellular stress responses by inhibiting the MAPK pathway, while *Serpina3f* is an important regulator of inflammatory responses [102,103]. Inhibiting this complex may relieve the inhibition of *Cacna1e* (a voltage-gated calcium channel), disrupt intracellular calcium signaling, and further affect *Esyt1* (extended synaptotagmin 1) that functions in lipid transport and cell membrane repair [104,105]. Our results show that upregulation of *Esyt1* is closely related to *Kcnk15* upregulation. Changes in intracellular calcium and lipid environments may serve as a feedback mechanism, upregulating *Kcnk15* to regulate membrane potential and ion balance in response to cellular stress [106,107,108]. While this regulatory cascade aligns with the established roles of calcium signaling and lipid metabolism in ion channel modulation, current transcriptional data only reveal associations; causal relationships require rigorous experimental validation.

Concurrently, increased *Kcnk15* expression levels raise the risk of membrane potential disturbance due to abnormal potassium efflux [109]. On another front, upregulation of dihydropyrimidinase-like 4 (*Dpysl4*) and downregulation of β-site APP cleaving enzyme 2 (*Bace2*) *may* synergistically elevate telethonin (*Tcap*), thereby inhibiting *Apoa4*, reducing the lipid order of pulmonary surfactant (PS), and increasing membrane fluidity [110,111]. Meanwhile, the moderate inhibitory effect of *miR-133a-3p* on *Apoa4* may persist. The dynamic balance of dual regulation shifts, and fat transport efficiency tends to become conservative [94,112,113], aligning with the species’ slow weight loss phenotype and conservative fat utilization strategy. This suggests a potential link between lipid metabolism and ion channel function, with *Esyt1* and *Kcnk15* as key nodes—though this remains a hypothetical regulatory network. Upregulation of *circ_0015576* may be a key upstream event, functioning as a molecular sponge for *miR-503-5p* [114]. By adsorbing *miR-503-5p*, *circ_0015576* may relieve the inhibition on *Kcnk15* translation, leading to increased expression levels [94]. The *circ_0015576*–*miR-503-5p*–*Kcnk15* ceRNA network integrates the regulatory effects of non-coding RNAs into the upregulation mechanisms of *Kcnk15.* However, the current evidence is restricted to expression patterns and predicted binding events, and further validation is required to elucidate the underlying regulatory mechanisms and confirm the causal relationships involved.

While this study has several acknowledged limitations, its findings nonetheless provide meaningful insights into lung adaptive responses to drought stress. First, bulk lung transcriptomic analysis lacks cell-type resolution, masking heterogeneous molecular responses across distinct cell populations. Second, inferences of ceRNA interactions, target binding and regulatory cascades rely heavily on existing literature and target gene functional annotations. Though these data link molecular changes to phenotypic outcomes, they reflect only correlative rather than mechanistic relationships, thereby limiting the depth of adaptive mechanistic interpretation. Third, the absence of histological analyses precludes direct visualization of structural and cellular remodeling in lung tissue—remodeling that may underpin the observed alterations in respiratory metabolism and transcriptional regulation during drought adaptation. Nevertheless, our results suggest that *O. sibirica* reduces overall energy expenditure under severe water scarcity, which may be associated with the transcriptional plasticity for suppressed lung cell proliferation, impaired pulmonary function and reduced gas exchange efficiency in lung tissue. These findings highlight a previously underappreciated dimension of systemic drought adaptation: the kidney is well established as the central organ governing water conservation, yet coordinated adaptive responses in other major organs (e.g., the lung) have received far less attention. Thus, this study offers novel perspectives for investigating multi-organ coordination in mediating physiological resilience to environmental stressors.

## 4. Materials and Methods

### 4.1. Animals

This study was conducted in accordance with the guidelines issued by the Animal Care and Treatment Ethics Committee of Inner Mongolia Agricultural University (NND2017012 and NND2022093). The healthy adult *O. sibirica* (average body weight = 101.5 ± 2.9 g) were captured using the cage-trapping method in the study area in May 2021. The study site is located in a typical desert region on the eastern edge of the Tengger Desert, Inner Mongolia, China (104°10′–105°30′ E, 37°24′–38°25′ N), characterized by an arid continental climate with low precipitation and high evaporation. The animals were transferred to the Desert Ecology and Rodent Pest Control Research Base at Inner Mongolia Agricultural University, where they were housed individually in custom-designed cages within a simulated natural environment. They were provided with standard rat pellet chow and water ad libitum under natural photoperiod conditions. Following capture, the animals underwent a two-week acclimatization and stabilization period: one week for recovery from handling stress and an additional week to achieve physiological stability. During this time, the food intake was recorded every two days, and the body weight was monitored daily until consistent values were obtained.

### 4.2. Experimental Designs

**Figure 6:**
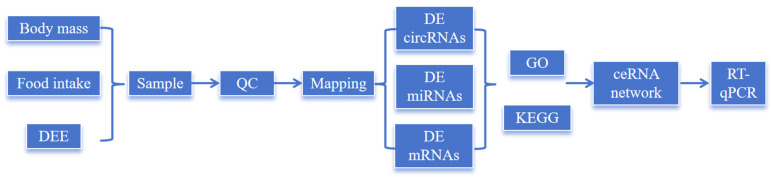
Technical roadmap.

*O. sibirica* were acclimated to the indoor environment for a two-week period, after which they were randomly assigned to one of two experimental groups: the control group (CK, n = 15, ♀9:♂6), which had ad libitum access to both food and water, and the water-deprivation stress group (WS, n = 14, ♀8:♂6), which received food ad libitum but was subjected to complete water deprivation throughout the 12-day experimental protocol. During the acclimation and experimental periods, a small number of animals died or were excluded from subsequent analyses due to individual health-related reasons. The slight imbalance in final group sizes (15 vs. 14) resulted from this non-treatment-related animal loss, with no mortality or exclusion attributable to the experimental procedures. Animals were maintained under their respective conditions for 12 days. At the end of the experimental protocol, the *O. sibirica* were anesthetized using 3.5% isoflurane for induction, followed by euthanasia via cervical dislocation after confirmation of unconsciousness. For small rodents weighing less than 200 g, cervical dislocation was performed by trained and experienced personnel. This euthanasia method was selected for its efficiency and minimal induction of distress in the animals. Lung tissues were immediately collected, rapidly frozen in liquid nitrogen, and stored at −80 °C until further analysis. The workflow of our current research is shown in Figure 6.

### 4.3. Measurement of Physiological Phenotypes

The body mass and food intake were measured every day during the acclimation period using an electronic balance accurate to ±0.01 g. The food intake was calculated based on the difference between food before and after a 24 h period. After each measurement, animals were provided with sufficient food.

The metabolic rate (oxygen consumption, VO_2_) of the animals was monitored using the Open Respiratory Metabolic System (Field Metabolic System, FMS, Sable Systems International, Las Vegas, NV, USA). The data were collected and processed using Expedata-P data analysis software v1.9.22 at an airflow rate of 0.75 L/min. The VO_2_ value was measured every 15 min. DEE was measured repeatedly in the same individuals at baseline (prior to water deprivation stress), following 6 days of stress exposure, and after 12 days of stress. DEE was assessed using repeated measures two-way ANCOVA, with body mass as a covariate to adjust for its confounding effect on energy expenditure. Prior to the DEE measurement, animals were acclimated in the metabolic chamber for 1 h to minimize handling- and chamber-related stress artifacts. The initial 1 h acclimation period was excluded from the analysis, and the subsequent continuous 24 h VO_2_ record was used to calculate DEE (mL O_2_·h^−1^). The environmental temperature was strictly maintained at 23 ± 0.5 °C, and the photoperiod was set to 16 h light:8 h dark (16L:8D). Food was provided ad libitum throughout the measurement; notably, water deprivation in the WS group was sustained during this period. The VO_2_ data from a continuous 24 h period were selected as the animal’s DEE (mL O_2_·h^−1^). The animals’ body mass and body temperature were recorded before and after the test.

### 4.4. Library Construction and Sequencing

Total RNA was extracted from individual samples, and RNA from three or four individuals was randomly pooled to generate composite samples. The lung tissue dataset comprised three biological replicates in the RNA-seq WS group and four in the CK group. All samples underwent rigorous quality assessment. RNA integrity and concentration were evaluated using the Agilent 2100 Bioanalyzer (Santa Clara, CA, USA). Following confirmation of the RNA quality, library construction was initiated by removing ribosomal RNA from total RNA, followed by fragmentation into 250–300 bp segments. First-strand cDNA synthesis was performed using fragmented RNA as a template and random oligonucleotide primers, followed by second-strand cDNA synthesis with dNTPs (dUTP, dATP, dGTP, and dCTP). The resulting double-stranded cDNA was purified and subsequently underwent end repair, A-tailing, and adapter ligation. cDNA fragments of approximately 350–400 bp were selected using AMPure XP (Beverly, MA, USA) beads. The second strand of cDNA containing uracil (U) was selectively degraded using USER enzyme, and the final library was amplified via PCR. The initial quantification was performed using Qubit fluorometry, and the libraries were normalized to 1 ng/μL. The insert size distribution was assessed on the Agilent 2100 Bioanalyzer, with expected peaks observed between 250 and 300 bp. Upon validation of the insert size, the effective molar concentration of each library was precisely determined through qPCR to ensure a concentration > 2 nM, thereby guaranteeing high-quality library preparation. Libraries that passed all quality control criteria were pooled according to their effective concentrations and sequencing depth requirements prior to paired-end 150 bp (PE150) sequencing on an Illumina platform (San Diego, CA, USA).

Total RNA was used as the starting material to analyze all three types of RNA, with a precise amount of 2 μg required specifically for circRNA analysis. For animal samples, ribosomal RNA (rRNA) was uniformly removed using the TruSeq Stranded Total RNA Library Prep Gold kit (Illumina, Cat. No. 20020599) (San Diego, CA, USA). For circRNA analysis, after rRNA removal, the residual components were purified through ethanol precipitation, followed by digestion of linear RNA with RNase R (Epicentre, Madison, WI, USA), at a ratio of 3 U per μg RNA to enrich circRNAs.

The NEBNext Ultra Directional RNA Library Prep Kit for Illumina (NEB E7420) was used for library construction. RNA was fragmented in the presence of divalent cations at high temperatures using the corresponding 5× reaction buffer. First-strand cDNA was synthesized using M-MuLV reverse transcriptase (with or without RNase H activity) with random hexamers as primers. Second-strand cDNA was then synthesized using DNA polymerase I and RNase H, with dUTP substituting dTTP in the circRNA reaction system. After end repair to generate blunt ends and adenylation of the 3′ ends, NEBNext hairpin adapters were ligated. Library fragments were purified using the AMPure XP system (Beverly, MA, USA), and fragments 370–420 bp in length were selected. Following the addition of 3 µL of USER enzyme (Ipswich, MA, USA), the mixture was incubated at 37 °C for 15 min and treated at 95 °C for 5 min. PCR amplification was performed using phusion high-fidelity DNA polymerase and corresponding primers, and the purified PCR products constituted the final library.

The NEBNext^®^ Multiplex Small RNA Library Prep Set for Illumina^®^ (NEB, Cat. No. E7300L) (Ipswich, MA, USA) was utilized. First, 3′ and 5′ adapters were ligated to both ends of small RNAs, respectively. First-strand cDNA was synthesized through hybridization with reverse transcription primers. After PCR amplification and purification, libraries were constructed by selecting insert fragments 18–40 bp in length.

All three types of libraries were quality-checked using the Agilent 5400 system (Santa Clara, CA, USA) and quantified to 1.5 nM through QPCR. Qualified libraries were pooled according to their effective concentrations and required data volume and then sequenced on the Illumina platform at Novogene (Beijing, China). The paired-end 150 bp (PE150) strategy was used for mRNA and circRNA sequencing, while the single-end 50 bp (SE50) strategy was employed for miRNA sequencing.

For mRNA and circRNA, raw FASTQ data were processed using self-developed Perl scripts to obtain clean reads by removing adapter-containing reads, poly-N-containing reads, and low-quality reads. Q20, Q30, and GC contents were calculated for quality assessment, and all downstream analyses were based on qualified clean reads (Supplementary Table S4). For miRNA, raw data were processed using self-developed Perl (5.30.0) and Python (3.9.0) scripts to generate clean reads by filtering out various unqualified reads. Q20, Q30, and GC contents were calculated, and sequences within a specific length range were selected for subsequent analyses. For animal samples, an rRNA proportion < 40% was used as the quality pass criterion (Supplementary Table S5).

For alignment of mRNA and circRNA, reference genome sequences and gene annotation files were downloaded first. The Hisat2 v2.0.5 software was used to build genome indexes and align paired-end clean reads. This software can construct a splice site database based on gene annotations, which improves the efficiency and accuracy of alignment (Supplementary Table S6). For the alignment of miRNA, Bowtie software (1.0.1) [115] was employed to align small RNA tags with reference sequences in a zero-mismatch mode. This alignment enabled the clarification of the expression levels and distribution characteristics of miRNAs (Supplementary Table S7).

StringTie v1.3.3b software was used to count the number of reads aligned to each mRNA gene, and FPKM (Fragments Per Kilobase of transcript per Million mapped reads) values were calculated. This metric corrects for both sequencing depth and transcript length, accurately reflecting the expression levels of mRNAs.

Normalization of circRNA expression was performed using the TPM (Transcripts Per Million) method [116]. The formula for normalization is as follows: Normalized expression level = (circRNA read count × 10^6^)/Library size (total circRNA read count of the sample). This method eliminates the impact of the differences in sequencing depth between samples. miRNA expression levels were normalized using the TPM method. The normalization formula is as follows: Normalized expression level = (miRNA-mapped read count/Total read count) × 10^6^. This normalization enables the comparison of miRNA expression levels across different samples.

Differential expression analysis was performed using edgeR v3.22.5 software. Statistical significance was assessed based on the *p*-value, with a threshold of |log_2_(fold change)| > 1 and adjusted *p*-value (padj) < 0.05 used to define significantly differentially expressed genes. Padj is the adjusted *p*-value derived from the Benjamini–Hochberg method for multiple testing correction, which controls the false discovery rate (FDR) when the risk of false positives is elevated.

### 4.5. Enrichment Analysis of GO and KEGG

GO and KEGG enrichment analyses of differentially expressed genes were conducted using clusterProfiler (3.8.1), with correction applied for gene length bias. GO terms with an adjusted *p*-value (padj) < 0.05 were considered significantly enriched. Selected significant biological functions and pathways were subsequently visualized using the online platform Microbiome (https://www.bioinformatics.com.cn, last accessed on 10 November 2025).

### 4.6. Network Construction

MicroRNA target sites within exonic regions of circRNA gene loci were identified using miRanda to determine miRNA binding sites on circRNAs [116,117]. The candidate miRNAs of interest were obtained by intersecting differentially expressed miRNAs with the predicted miRNA targets. Downstream target genes of these miRNAs were predicted using the miRanda software (v3.3a) in *O. sibirica*. The prediction was performed under stringent filtering criteria to ensure high reliability: a score cutoff of 140, an energy cutoff [75] (-en) of −10 kcal/mol for miRNA-mRNA duplex binding, a scaling factor (-scale) of 4, and enforcement of strict seed region pairing (-strict). The predicted target genes were then intersected with experimentally derived differentially expressed mRNAs from sequencing data to identify overlapping differentially expressed mRNA targets. Finally, a competing endogenous RNA (ceRNA) network was constructed using Cytoscape 3.8.0.

### 4.7. Lung Gene Expression Detected Through Real-Time Quantitative PCR (RT-qPCR)

RT-qPCR experiments were performed as follows: cDNA samples (1 μL) served as templates for amplification with gene-specific primers (Supplementary Table S8). Reverse transcription was conducted using the TUREscript 1st Strand cDNA Synthesis Kit (PC1802, Adlai, Township, NJ, USA), and fluorescent detection utilized 2 × SYBR^®^ Green MIX (PC3302, Adlai). The 20 μL reaction system contained 5 μL 2 × SYBR^®^ Green Supermix, 0.5 μL forward primer, 0.5 μL reverse primer, 1 μL cDNA, and 3 μL RNase-free ddH_2_O. Thermal cycling included initial polymerase activation at 95 °C for 3 min, 39 cycles of 95 °C for 10 s and 60 °C for 30 s (with plate read), and melting curve analysis (60–95 °C, 1 °C/cycle, 4 s hold) for primer specificity validation. Relative gene expression levels were determined using the 2^−^ΔΔCt method [118], based on three biological replicates in the WS group and four in the CK group.

Designed and optimized using Beacon Designer 7.9, mRNA primers (18–22 bp, Tm 56–63.8 °C, GC 45–55.6%) showed no significant hairpin/self-dimer/cross-dimer formation (free energy ≤ −1.5 kcal/mol). Melting curve analysis confirmed single sharp peaks (no non-specific amplification/primer dimers) and consistent peak temperatures (variation ≤ 2 °C) across samples. The primer efficiency (95–105%) was validated by stable Cq values (Std. Dev ≤ 1.053) and consistent design parameters.

Stem-loop RT-PCR-designed miRNA primers ensured specificity for mature miRNAs: RT primers contained a universal stem-loop sequence (GTCGTATCCAGTGCAGGGTCCGAGGTATTCGCACTGGATACGAC) fused to the first 6 bases of miRNA reverse complements, with forward primers targeting mature sequences and reverse primers recognizing conserved stem-loop regions. Optimized via Primer Premier (16–20 bp, Tm 53.9–58.8 °C, GC 45–61.1%), primers lacked hairpin/non-specific dimers. Melting curve analysis showed single sharp peaks (variation ≤ 2 °C), and the efficiency (95–105%) was supported by stable Cq values (Std. Dev ≤ 1.370) and uniform amplification curves.

circRNA primers targeted back-splice junctions of *novel_circ_0024991*, *novel_circ_0019806*, and *circ_0007222* (18–22 bp, Tm 56.92–62.2 °C, GC 45–55.56%), ensuring specificity for circular transcripts over linear mRNA/genomic DNA. Optimized via Primer Premier (no hairpin/non-specific dimers), the specificity was confirmed by melting curve analysis (single sharp peaks) and Primer-BLAST (no off-target binding in *O.sibirica*) (https://www.ncbi.nlm.nih.gov/tools/primer-blast/, last accessed on 1 September 2025). Efficiency (95–105%) was validated by stable Cq values (Std. Dev ≤ 0.709). The initial primers for novel_circ_0024991 were reoptimized to an alternative back-splice junction, improving amplification stability (Cq range: 28.46–30.60).

### 4.8. Statistical Analyses

Statistical analyses and data visualization were performed using IBM SPSS Statistics 26, GraphPad Prism 9.0.1, and R 4.2.2, with data presented as the mean ± standard error (Mean ± SE). For physiological and qPCR data, the normality and homogeneity of variances were first assessed using Kolmogorov–Smirnov and Levene’s tests. Data not conforming to these assumptions were analyzed using the Kruskal–Wallis H non-parametric test. DEE was assessed through repeated measures two-way ANCOVA. In the repeated measures ANCOVA, the sphericity assumption was verified through the Mauchly test. If the assumption was violated, Greenhouse–Geisser and Huynh–Feldt corrections were applied. RT-qPCR data were analyzed using the *t*-test. Statistical significances were set at *p* < 0.05 for significant differences and *p* < 0.01 for highly significant differences. To control the family-wise error rate and mitigate the risk of false-positive inferences arising from multiple testing, the Bonferroni correction was rigorously applied to all post hoc pairwise comparisons of RT-qPCR data (key RNA expression), body mass, food intake, and DEE. Specifically, in the repeated-measures analysis, 12 pairwise comparisons were conducted for one physiological index (e.g., DEE across time points and groups), whereas 6 pairwise comparisons were performed for each of the other two indices (e.g., body mass and food intake across groups at a given time point). Adjusted *p*-values—derived by dividing the nominal α-level (0.05) by the respective number of comparisons—were used to determine statistical significance for each comparison. Pathway plots were generated using Adobe Illustrator 2023. Transcriptome and RT-qPCR data were judged for outliers using the IQR method. No data exceeded the range of 1.5× IQR, and no samples were excluded.

## 5. Conclusions

Under simulated extreme drought via water deprivation, *O. sibirica* exhibited continuous body mass decline, a stage-wise reduction in food intake, and a 12.6% lower DEE during the active period compared to the control group. Whole-transcriptome analysis identified 55 differentially expressed circRNAs, 15 differentially expressed miRNAs, and 554 differentially expressed mRNAs. The constructed ceRNA network contained two circRNAs, three miRNAs, and four mRNAs. The candidate ceRNA axis “*circ_0015576*–*miR-503-5p*–*Kcnk15*” may alleviate the post-transcriptional inhibition of *Kcnk15* by *miR-503-5p*, and participate in the regulation of lung tissue structure, cell cycle progression, and electrolyte balance. This study reveals a potential strategy for *O. sibirica* to adapt to drought via “active energy regulation–molecular network synergy,” providing a reference framework for investigating drought adaptation in non-renal organs of small rodents and offering insights for species conservation in arid regions. Notably, this study is observational and predictive; the causal relationships of the core ceRNA axis and its potential applications in human-related diseases require confirmation through subsequent functional experiments and translational research.

## Figures and Tables

**Table 1:** circRNA and miRNA binding ability and coding potential prediction.

miRNA ID	circRNA ID	Prediction of Targeting Relationships: Total Scoring Values Across All Binding Sites	Total Energy Value
miR-503-5p	circ_0015576	153	−25.84
miR-133a-3p	circ_0027390	140	−12.94
miR-133b-3p	circ_0027390	140	−12.94

## Data Availability

All genomic sequencing data generated in this project are available through the NCBI BioProject under accession number PRJNA1181308. However, raw whole-transcriptome sequencing data are temporarily restricted from public release due to ongoing related research.

## Supplementary Materials

The following supporting information can be downloaded at https://www.mdpi.com/article/10.3390/ijms27031458/s1.

## Abbreviations

The following abbreviations are used in this manuscript:

*Acan*	aggrecan
*Agmo*	alkylglycerol monooxygenase
*Aldh8a1*	aldehyde dehydrogenase 8 family member a1
*Alox15*	arachidonate 15-lipoxygenase
*Aoc1*	amine oxidase, copper containing 1
*ApoA4*	apolipoprotein A-IV
*Aqp2*	aquaporin 2
*Bace2*	β-site APP cleaving enzyme 2
BP	biological process
CC	cellular component
MF	molecular function
WS	water-restriction stress
CK	control conditions
GO	Gene Ontology
KEGG	Kyoto Encyclopedia of Genes and Genomes
ADMR	average daily metabolic rate
BMR	basal metabolic rate
DEE	daily energy expenditure
*Cacna1e*	calcium voltage-gated channel subunit alpha1 E
*Ccnb1*	cyclin B1
ceRNA	competing endogenous RNA
circRNAs	circular RNA
miRNAs	microRNAs
CNCI	Coding-Non-Coding Index
CPC	Coding Potential Calculator
DE	differentially expressed
DEG	differentially expressed gene
*Dpysl4*	dihydropyrimidinase-like 4
*Dusp15*	dual-specificity phosphatase 15
ECM	extracellular matrix
*Ehhadh*	enoyl-CoA hydratase and 3-hydroxyacyl-CoA dehydrogenase
*Eln*	elastin
*EMT*	epithelial–mesenchymal transition
*Esyt1*	extended synaptotagmin 1
*EWL*	evaporative water loss
GC	guanine–cytosine content
*Golgb1*	golgin b1
*Il-6*	interleukin-6
IPCC	Intergovernmental Panel on Climate Change
*IPF*	idiopathic pulmonary fibrosis
*Kcnk15*	potassium two-pore domain channel subfamily K member 15
MAPK	mitogen-activated protein kinase
*Mybl2*	myb proto-oncogene like 2
*Myh7*	muscle fiber structure genes myosin heavy chain 7
*NF-κβ*	nuclear factor-κβ
*Pi16*	peptidase inhibitor 16
*PS*	pulmonary surfactant
QC	quality control
RISC	RNA-induced silencing complex
RMT	relative medullary thickness
ROS	reactive oxygen species
RT-qPCR	reverse transcription quantitative polymerase chain reaction
*Ryr3*	ryanodine receptor 3
*Scd1*	stearoyl-CoA desaturase 1
*Scnn1a*	sodium channel, nonvoltage-gated 1 alpha subunit
*Serpina3f*	serine protease inhibitor a3f
*Smad*	sma- and mad-related proteins
SNP	single nucleotide polymorphism
*Tcap*	telethonin
*Tnf-α*	tumor necrosis factor-α
*Tnni1*	troponin I type 1
*Tnnt2*	troponin T type 2
*TPM*	transcripts per million
*Trpv5*	transient receptor potential vanilloid 5
*Tspan10*	tetraspanin 10
*Umod*	uromoduli
