# Supplementary material for: Metabolic Adaptation and Pulmonary ceRNA Network Plasticity in Orientallactaga sibirica During Water Deprivation Stress
Source: Int J Mol Sci. 2026 Feb 1;27(3):1458. doi: 10.3390/ijms27031458 (PMC12897892; doi:10.3390/ijms27031458)
Supplement: Supplementary file 1 [file ijms-27-01458-s001.zip › ijms-4074073-supplementary.pdf]

Supplementary material

Supplementary Table S1. The Gene Ontology (GO) enrichment analysis of differential expressed mRNAs.

GOID	Category	Description	padj
GO:0016042	BP	lipid catabolic process	0.000286166694826615
GO:0005520	MF	insulin-like growth factor binding	0.000371529178930188
GO:0019838	MF	growth factor binding	0.000546462855886465
GO:0008289	MF	lipid binding	0.00146181097899603
GO:0055114	BP	oxidation-reduction process	0.00242367718154702
GO:0032787	BP	monocarboxylic acid metabolic process	0.00284794464528737
GO:0016810	MF	hydrolase activity, acting on carbon-nitrogen (but not peptide) bonds	0.00305563701837732
GO:0044427	CC	chromosomal part	0.00321819723117223
GO:0070588	BP	calcium ion transmembrane transport	0.00459726288236167
GO:0099080	CC	supramolecular complex	0.00472506241188179
GO:0099081	CC	supramolecular polymer	0.00472506241188179
GO:0099512	CC	supramolecular fiber	0.00472506241188179
GO:0005507	MF	copper ion binding	0.0058753652303442
GO:0016702	MF	oxidoreductase activity, acting on single donors with incorporation of molecular oxygen, incorporation of two atoms of oxygen	0.0058753652303442
GO:0005509	MF	calcium ion binding	0.00588579890903464
GO:0006816	BP	calcium ion transport	0.00642027482645921
GO:0015629	CC	actin cytoskeleton	0.00688125988312506
GO:0016491	MF	oxidoreductase activity	0.00693727257005478
GO:0016701	MF	oxidoreductase activity, acting on single donors with incorporation of molecular oxygen	0.00752226179513951
GO:0007155	BP	cell adhesion	0.00848729250138339
GO:0022610	BP	biological adhesion	0.00848729250138339
GO:0005694	CC	chromosome	0.0088178761696426
GO:0051213	MF	dioxygenase activity	0.00947762824860346

GO:0009308	BP	amine metabolic process	0.0105683300052422
GO:0000775	CC	chromosome, centromeric region	0.0123222023015968
GO:0005262	MF	calcium channel activity	0.0133608733948718
GO:0033043	BP	regulation of organelle organization	0.0135121249266126
GO:0046983	MF	protein dimerization activity	0.0137871274531831
GO:0008017	MF	microtubule binding	0.0146582481774313
GO:0046982	MF	protein heterodimerization activity	0.0146805465450656
GO:0016032	BP	viral process	0.0160638062083535
GO:0044403	BP	symbiont process	0.0160638062083535
GO:0098687	CC	chromosomal region	0.0182805410629557
GO:0008064	BP	regulation of actin polymerization or depolymerization	0.0187126210606809
GO:0008154	BP	actin polymerization or depolymerization	0.0187126210606809
GO:0030041	BP	actin filament polymerization	0.0187126210606809
GO:0030832	BP	regulation of actin filament length	0.0187126210606809
GO:0030833	BP	regulation of actin filament polymerization	0.0187126210606809
GO:0032271	BP	regulation of protein polymerization	0.0187126210606809
GO:0032535	BP	regulation of cellular component size	0.0187126210606809
GO:0032956	BP	regulation of actin cytoskeleton organization	0.0187126210606809
GO:0032970	BP	regulation of actin filament-based process	0.0187126210606809
GO:0043254	BP	regulation of protein complex assembly	0.0187126210606809
GO:0110053	BP	regulation of actin filament organization	0.0187126210606809
GO:1902903	BP	regulation of supramolecular fiber organization	0.0187126210606809
GO:0019058	BP	viral life cycle	0.0195686736598857
GO:0051493	BP	regulation of cytoskeleton organization	0.0195686736598857

GO:0016684	MF	oxidoreductase activity, acting on peroxide as acceptor	0.0209331267532725
GO:0000785	CC	chromatin	0.021033206335018
GO:0005539	MF	glycosaminoglycan binding	0.0210600939147899
GO:0044255	BP	cellular lipid metabolic process	0.0227888619148989
GO:0044087	BP	regulation of cellular component biogenesis	0.0232504902660537
GO:0044419	BP	interspecies interaction between organisms	0.0232976566247817
GO:0005544	MF	calcium-dependent phospholipid binding	0.02401438474465
GO:0004222	MF	metalloendopeptidase activity	0.024033528572316
GO:0005975	BP	carbohydrate metabolic process	0.0245116306894712
GO:0016209	MF	antioxidant activity	0.0248291826363829
GO:0044430	CC	cytoskeletal part	0.0253165006361235
GO:0006629	BP	lipid metabolic process	0.0267907689674235
GO:0090066	BP	regulation of anatomical structure size	0.0284428452151471
GO:0051128	BP	regulation of cellular component organization	0.0296854394441194
GO:0000786	CC	nucleosome	0.0299879220395302
GO:0048037	MF	cofactor binding	0.0300837468555113
GO:0003777	MF	microtubule motor activity	0.0303561280354781
GO:0005540	MF	hyaluronic acid binding	0.0306495676431954
GO:0015085	MF	calcium ion transmembrane transporter activity	0.0311826921679556
GO:0015631	MF	tubulin binding	0.0314380491139116
GO:0032993	CC	protein-DNA complex	0.0329281250309576
GO:0006631	BP	fatty acid metabolic process	0.0332839901794741
GO:0005179	MF	hormone activity	0.0340040107585347
GO:0003774	MF	motor activity	0.0341408864053512
GO:0044815	CC	DNA packaging complex	0.0360673867773652
GO:0015698	BP	inorganic anion transport	0.0378342661761815
GO:0005044	MF	scavenger receptor activity	0.0393140959601833
GO:0038024	MF	cargo receptor activity	0.0393140959601833
GO:0065008	BP	regulation of biological quality	0.0429265639382736
GO:0006928	BP	movement of cell or subcellular component	0.0436467597352227
GO:0006817	BP	phosphate ion transport	0.046874457575166
GO:0018149	BP	peptide cross-linking	0.046874457575166

GO:0030258	BP	lipid modification	0.046874457575166
GO:0044242	BP	cellular lipid catabolic process	0.046874457575166
GO:0051301	BP	cell division	0.046874457575166
GO:0006979	BP	response to oxidative stress	0.0481608756092954
GO:0007015	BP	actin filament organization	0.0481608756092954
GO:0005102	MF	signaling receptor binding	0.0483173512595828
GO:0044421	CC	extracellular region part	0.0492015828580431
GO:0008237	MF	metallopeptidase activity	0.0493296818083592

Supplementary Table S2. The Kyoto Encyclopedia of Genes and Genomes (KEGG) analysis of differential expressed mRNAs.

KEGGID	Description	padj
rno00515	Mannose type O-glycan biosynthesis	0.693332137
rno04151	PI3K-Akt signaling pathway	0.000291607309516338
rno04114	Oocyte meiosis	0.000378246928097052
rno05206	MicroRNAs in cancer	0.000490666114825491
rno00480	Glutathione metabolism	0.000581747998584201
rno04216	Ferroptosis	0.000807606108998387
rno01212	Fatty acid metabolism	0.000911359891927487
rno05224	Breast cancer	0.00238182535276841
rno04142	Lysosome	0.00285122922298348
rno04970	Salivary secretion	0.00303019654301005
rno04218	Cellular senescence	0.00325079593411147
rno05203	Viral carcinogenesis	0.00328313903660908
rno05218	Melanoma	0.00526129588397585
rno05214	Glioma	0.0053286377391216
rno04964	Proximal tubule bicarbonate reclamation	0.00548390991194641
rno04152	AMPK signaling pathway	0.00650762206544326
rno04972	Pancreatic secretion	0.00691991405659251
rno04971	Gastric acid secretion	0.00730923033890185
rno00380	Tryptophan metabolism	0.00780999610088557
rno05222	Small cell lung cancer	0.0079242387231622
rno04068	FoxO signaling pathway	0.00871694797220744
rno04611	Platelet activation	0.0101138609174579
rno05143	African trypanosomiasis	0.0102309425134439
rno00061	Fatty acid biosynthesis	0.0117298268709387
rno04070	Phosphatidylinositol signaling system	0.0123170781747943
rno04934	Cushing syndrome	0.0141664042632649
rno01040	Biosynthesis of unsaturated fatty acids	0.0146064668212034
rno01522	Endocrine resistance	0.0163505684204189
rno04979	Cholesterol metabolism	0.0165961123210848
rno04918	Thyroid hormone synthesis	0.0178836860092657
rno04726	Serotonergic synapse	0.0191547989598317
rno04010	MAPK signaling pathway	0.0198178662038362
rno04115	p53 signaling pathway	0.0224773314510114
rno04146	Peroxisome	0.0245253537097461
rno00330	Arginine and proline metabolism	0.0262403302793681
rno04730	Long-term depression	0.0267089587707346
rno04260	Cardiac muscle contraction	0.0272315733726227

rno05146	Amoebiasis	0.0275999422039794
rno05144	Malaria	0.0332849969539442
rno04928	Parathyroid hormone synthesis, secretion and action	0.0360733318563821
rno00860	Porphyrin metabolism	0.037401774492798
rno00591	Linoleic acid metabolism	0.0423676116621768
rno00100	Steroid biosynthesis	0.0439865305556672
rno05215	Prostate cancer	0.0483301141050841
rno04613	Neutrophil extracellular trap formation	0.0494145651671779

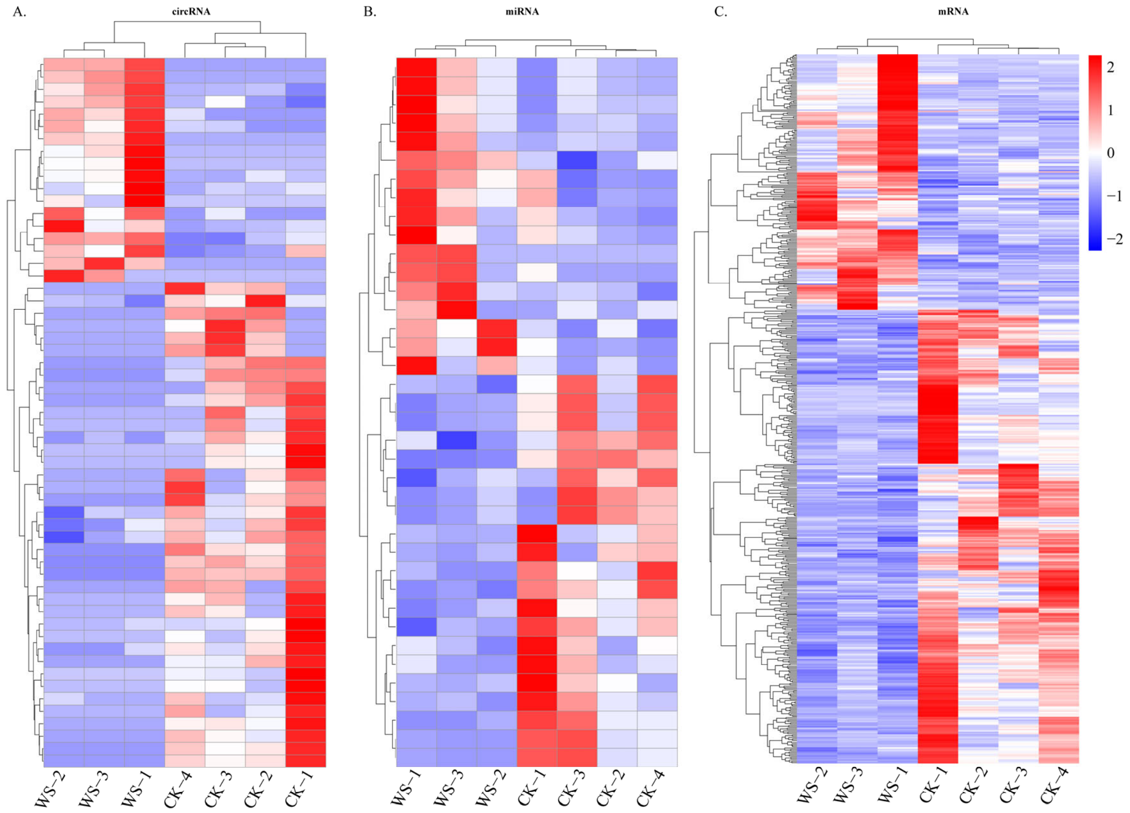

Supplementary Figure S1. Differential expression of RNAs (A-C) The differences of circRNAs, miRNAs, and mRNA between water deprivation stress (WS) versus control (CK) groups were shown by heat map. High expression is shown in red and low expression in blue.

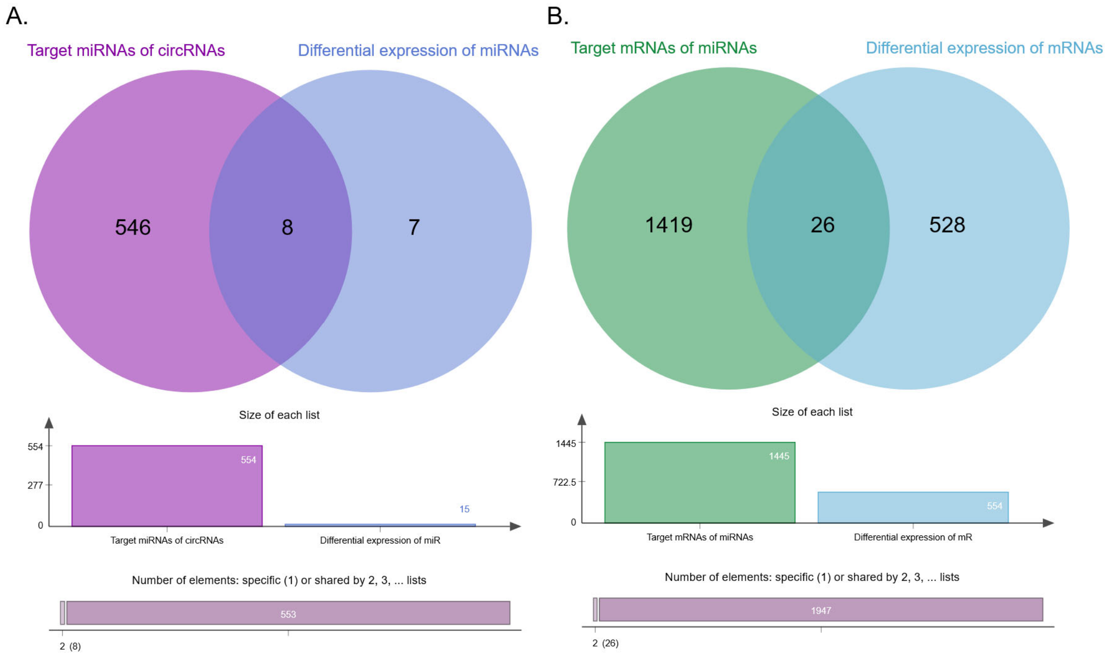

Supplementary Figure S2. The connections between circRNA, miRNA and mRNA (A) Purple represents miRNAs that are capable of spongy circRNAs predicted by CircInteractome, blue-violet represents the differential miRNAs obtained by RNA sequencing (padj<0.05), and the overlaps represent the differentially expressed miRNAs that we ultimately need. (B) The green circle represents the predicted mRNA that can target all miRNAs, and the blue circles represent the intersection of each miRNA's predicted and actual sequencing results.

Supplementary Table S3. Correlational analysis of circRNA-miRNA-mRNA regulatory networks for *O.sibirica*.

RNAs ID	Type of target molecule	Target molecule ID	Spearman	padj
circ_0014702	mRNA	Dusp15	-0.964	0
circ_0014702	mRNA	Serpina3f	0.935	0.045
circ_0014702	mRNA	Cacna1e	0.927	0.045
circ_0014702	mRNA	Tspan10	0.927	0.045
circ_0014702	mRNA	Znf420	-0.927	0.045
circ_0015576	mRNA	Dusp15	0.906	0.045
circ_0015576	mRNA	Znf585a	0.906	0.045
circ_0027390	mRNA	Ccnb1	0.927	0.045
circ_0027390	mRNA	Golgb1	0.927	0.045
circ_0027390	mRNA	Znf585b	0.927	0.045
circ_0007222	miRNA	miR-133a-3p	-0.906	0.045
circ_0007222	miRNA	miR-133c	-0.906	0.045
circ_0007222	miRNA	miR-133a-5p	-0.906	0.045
circ_0014702	miRNA	miR-133a-5p	-0.927	0.045
circ_0015576	miRNA	miR-503-5p	-0.906	0.045
circ_0015576	miRNA	miR-133a-5p	0.906	0.045
circ_0015576	miRNA	miR-135a-5p	0.906	0.045
circ_0027390	miRNA	miR-135a-5p	-0.927	0.045
circ_0015576	mRNA	Kcnk15	0.749	0.045
circ_0014702	mRNA	Ccnb1	0.889	0.049304
circ_0014702	mRNA	Golgb1	0.889	0.049304
circ_0014702	mRNA	Znf585b	0.889	0.049304
circ_0027390	mRNA	Dpysl4	-0.889	0.049304
circ_0014702	miRNA	miR-135a-5p	-0.889	0.049304
circ_0007222	mRNA	Pi16	0.875	0.054
circ_0007222	mRNA	Dusp15	-0.867	0.054
circ_0014702	mRNA	Znf850	0.885	0.054
circ_0014702	mRNA	Apoa4	0.852	0.054
circ_0015576	mRNA	Cacna1e	-0.867	0.054
circ_0015576	mRNA	Dpysl4	0.867	0.054
circ_0015576	mRNA	Golgb1	-0.867	0.054
circ_0015576	mRNA	Tspan10	-0.867	0.054
circ_0015576	mRNA	Znf420	0.867	0.054
circ_0015576	mRNA	Znf850	-0.859	0.054
circ_0024991	mRNA	Esyt1	0.87	0.054
circ_0024991	mRNA	Dpysl4	-0.867	0.054
circ_0024991	mRNA	Znf585a	-0.867	0.054

circ_0027390	mRNA	Mybl2	0.86	0.054
circ_0027390	mRNA	Serpina3f	0.86	0.054
circ_0027390	mRNA	Cacna1e	0.852	0.054
circ_0027390	mRNA	Tspan10	0.852	0.054
circ_0027390	mRNA	Znf420	-0.852	0.054
circ_0027390	mRNA	Znf585a	-0.852	0.054
circ_0014702	miRNA	miR-133a-3p	-0.852	0.054
circ_0014702	miRNA	miR-133c	-0.852	0.054
circ_0027390	miRNA	miR-133a-5p	-0.852	0.054
circ_0027390	mRNA	Znf850	0.846	0.056348
circ_0015576	mRNA	Serpina3f	-0.835	0.065489
circ_0019806	mRNA	Znf585b	0.821	0.077625
circ_0007222	mRNA	Ccnb1	0.808	0.081
circ_0007222	mRNA	Golgb1	0.808	0.081
circ_0007222	mRNA	Znf585b	0.808	0.081
circ_0027390	mRNA	Apoa4	0.815	0.081
circ_0027390	mRNA	Znf268	0.815	0.081
circ_0007222	miRNA	miR-135a-5p	-0.808	0.081
circ_0015576	miRNA	miR-133a-3p	0.808	0.081
circ_0015576	miRNA	miR-133c	0.808	0.081
circ_0015576	mRNA	Mybl2	-0.795	0.093789
circ_0015576	mRNA	Apoa4	-0.788	0.096102
circ_0015576	mRNA	Ccnb1	-0.788	0.096102
circ_0014702	mRNA	Pi16	0.785	0.0972
circ_0014702	mRNA	Tcap	0.778	0.100286
circ_0027390	mRNA	Dusp15	-0.778	0.100286
circ_0014702	miRNA	miR-133b-3p	-0.778	0.100286
circ_0007222	mRNA	Serpina3f	0.775	0.103304
circ_0007222	mRNA	Cacna1e	0.768	0.103304
circ_0007222	mRNA	Tspan10	0.768	0.103304
circ_0007222	mRNA	Znf420	-0.768	0.103304
circ_0007222	miRNA	miR-133b-3p	-0.768	0.103304
circ_0015576	miRNA	miR-135b-5p	0.768	0.103304
circ_0019806	mRNA	Pi16	0.757	0.1134
circ_0007222	mRNA	Tcap	0.749	0.116027
circ_0015576	mRNA	Znf585b	-0.749	0.116027
circ_0019806	mRNA	Ccnb1	0.75	0.116027
circ_0014702	mRNA	Znf585a	-0.741	0.1215
circ_0027390	mRNA	Kcnk15	-0.741	0.1215
circ_0007222	mRNA	Znf585a	-0.729	0.130846
circ_0015576	mRNA	Tcap	-0.729	0.130846

circ_0019806	miRNA	miR-133a-5p	-0.714	0.145595
circ_0014702	mRNA	Kcnk15	-0.704	0.1485
circ_0014702	miRNA	miR-135b-5p	-0.704	0.1485
circ_0015576	miRNA	miR-133b-3p	0.709	0.1485
circ_0027390	miRNA	miR-133a-3p	-0.704	0.1485
circ_0027390	miRNA	miR-133c	-0.704	0.1485
circ_0015576	mRNA	Esyt1	-0.685	0.171529
circ_0019806	mRNA	Znf585a	-0.679	0.176087
circ_0019806	mRNA	Esyt1	0.67	0.176087
circ_0024991	mRNA	Mybl2	0.676	0.176087
circ_0024991	mRNA	Ccnb1	0.67	0.176087
circ_0024991	mRNA	Golgb1	0.67	0.176087
circ_0024991	mRNA	Znf585b	0.67	0.176087
circ_0024991	miRNA	miR-135a-5p	-0.67	0.176087
circ_0014702	miRNA	miR-503-5p	0.667	0.177677
circ_0019806	mRNA	Dusp15	-0.643	0.196714
circ_0019806	mRNA	Golgb1	0.643	0.196714
circ_0019806	mRNA	Tcap	0.643	0.196714
circ_0019806	miRNA	miR-133a-3p	-0.643	0.196714
circ_0019806	miRNA	miR-133c	-0.643	0.196714
circ_0014702	mRNA	Dpysl4	-0.63	0.199029
circ_0015576	mRNA	Eln	-0.63	0.199029
circ_0015576	mRNA	Znf268	-0.63	0.199029
circ_0019806	mRNA	Serpina3f	0.631	0.199029
circ_0027390	mRNA	Esyt1	0.634	0.199029
circ_0024991	miRNA	miR-133a-5p	-0.63	0.199029
circ_0027390	miRNA	miR-503-5p	0.63	0.199029
circ_0007222	mRNA	Znf850	0.613	0.2175
circ_0015576	mRNA	Bace2	-0.611	0.2175
circ_0024991	mRNA	Znf268	0.611	0.2175
circ_0019806	miRNA	miR-135a-5p	-0.607	0.219963
circ_0014702	mRNA	Mybl2	0.598	0.229745
circ_0007222	mRNA	Apoa4	0.591	0.236432
circ_0019806	mRNA	Cacna1e	0.571	0.253565
circ_0019806	mRNA	Tspan10	0.571	0.253565
circ_0019806	mRNA	Znf420	-0.571	0.253565
circ_0024991	miRNA	miR-503-5p	0.571	0.253565
circ_0014702	mRNA	Znf268	0.556	0.27
circ_0027390	miRNA	miR-133b-3p	-0.556	0.27
circ_0019806	mRNA	Apoa4	0.536	0.295169
circ_0019806	mRNA	Znf850	0.519	0.31455

circ_0027390	mRNA	Bace2	0.519	0.31455
circ_0024991	mRNA	Kcnk15	-0.512	0.321322
circ_0019806	mRNA	Kcnk15	-0.5	0.330328
circ_0024991	mRNA	Serpina3f	0.497	0.330328
circ_0024991	mRNA	Cacna1e	0.493	0.330328
circ_0024991	mRNA	Tspan10	0.493	0.330328
circ_0024991	mRNA	Znf420	-0.493	0.330328
circ_0024991	miRNA	miR-133a-3p	-0.493	0.330328
circ_0024991	miRNA	miR-133c	-0.493	0.330328
circ_0007222	mRNA	Esyt1	0.489	0.332791
circ_0014702	mRNA	Bace2	0.482	0.33884
circ_0027390	mRNA	Tcap	0.482	0.33884
circ_0007222	mRNA	Znf268	0.473	0.34697
circ_0014702	mRNA	Esyt1	0.47	0.34697
circ_0024991	mRNA	Znf850	0.47	0.34697
circ_0019806	mRNA	Dpysl4	-0.464	0.3528
circ_0007222	mRNA	Dpysl4	-0.453	0.360391
circ_0024991	mRNA	Apoa4	0.453	0.360391
circ_0024991	mRNA	Dusp15	-0.453	0.360391
circ_0027390	miRNA	miR-135b-5p	-0.445	0.369453
circ_0024991	mRNA	Bace2	0.433	0.37762
circ_0007222	miRNA	miR-503-5p	0.433	0.37762
circ_0007222	miRNA	miR-135b-5p	-0.433	0.37762
circ_0015576	mRNA	Pi16	-0.427	0.381375
circ_0019806	miRNA	miR-503-5p	0.429	0.381375
circ_0027390	mRNA	Pi16	0.411	0.40109
circ_0014702	mRNA	Eln	0.408	0.40389
circ_0007222	mRNA	Mybl2	0.398	0.415469
circ_0019806	mRNA	Bace2	0.393	0.416416
circ_0019806	miRNA	miR-133b-3p	-0.393	0.416416
circ_0007222	mRNA	Kcnk15	-0.374	0.44064
circ_0027390	mRNA	Eln	0.371	0.443086
circ_0024991	mRNA	Tcap	0.335	0.493461
circ_0019806	miRNA	miR-135b-5p	-0.321	0.510353
circ_0019806	mRNA	Mybl2	0.252	0.61539
circ_0019806	mRNA	Znf268	0.25	0.6156
circ_0024991	miRNA	miR-133b-3p	-0.217	0.665654
circ_0007222	mRNA	Bace2	0.158	0.749887
circ_0024991	mRNA	Eln	0.158	0.749887
circ_0024991	miRNA	miR-135b-5p	-0.158	0.749887
circ_0024991	mRNA	Pi16	0.109	0.825188

circ_0019806	mRNA	Eln	-0.071	0.88446
circ_0007222	mRNA	Eln	0.02	0.967
miR_133a_5p	mRNA	Dusp15	0.964	0
miR_135a_5p	mRNA	Golgb1	-0.964	0
miR_133a_3p	mRNA	Dusp15	0.929	0.003
miR_133a_5p	mRNA	Golgb1	-0.929	0.003
miR_133c	mRNA	Dusp15	0.929	0.003
miR_135a_5p	mRNA	Cacna1e	-0.929	0.003
miR_135a_5p	mRNA	Dusp15	0.929	0.003
miR_135a_5p	mRNA	Tspan10	-0.929	0.003
miR_135a_5p	mRNA	Znf420	0.929	0.003
miR_135a_5p	mRNA	Serpina3f	-0.901	0.006
miR_133a_5p	mRNA	Cacna1e	-0.893	0.007
miR_133a_5p	mRNA	Tspan10	-0.893	0.007
miR_133a_5p	mRNA	Znf420	0.893	0.007
miR_133a_5p	mRNA	Znf585a	0.893	0.007
miR_133b_3p	mRNA	Dusp15	0.893	0.007
miR_135a_5p	mRNA	Ccnb1	-0.893	0.007
miR_133a_5p	mRNA	Serpina3f	-0.865	0.012
miR_133a_5p	mRNA	Ccnb1	-0.857	0.014
miR_135a_5p	mRNA	Znf585a	0.857	0.014
miR_135a_5p	mRNA	Znf585b	-0.857	0.014
miR_135a_5p	mRNA	Znf850	-0.852	0.015
miR_135a_5p	mRNA	Mybl2	-0.847	0.016
miR_133a_3p	mRNA	Golgb1	-0.821	0.023
miR_133a_3p	mRNA	Znf585a	0.821	0.023
miR_133a_5p	mRNA	Znf585b	-0.821	0.023
miR_133c	mRNA	Golgb1	-0.821	0.023
miR_133c	mRNA	Znf585a	0.821	0.023
miR_135a_5p	mRNA	Znf268	-0.821	0.023
miR_133a_3p	mRNA	Cacna1e	-0.786	0.036
miR_133a_3p	mRNA	Tspan10	-0.786	0.036
miR_133a_3p	mRNA	Znf420	0.786	0.036
miR_133c	mRNA	Cacna1e	-0.786	0.036
miR_133c	mRNA	Tspan10	-0.786	0.036
miR_133c	mRNA	Znf420	0.786	0.036
miR_135a_5p	mRNA	Apoa4	-0.786	0.036
miR_135a_5p	mRNA	Dpysl4	0.786	0.036
miR_503_5p	mRNA	Dpysl4	-0.786	0.036
miR_503_5p	mRNA	Znf585a	-0.786	0.036
miR_133a_5p	mRNA	Znf850	-0.778	0.039

miR_503_5p	mRNA	Kcnk15	-0.75	0.049
miR_133a_5p	mRNA	Tcap	-0.75	0.052
miR_133b_3p	mRNA	Cacna1e	-0.75	0.052
miR_133b_3p	mRNA	Tspan10	-0.75	0.052
miR_133b_3p	mRNA	Znf420	0.75	0.052
miR_135b_5p	mRNA	Dusp15	0.75	0.052
miR_503_5p	mRNA	Bace2	0.75	0.052
miR_503_5p	mRNA	Eln	0.75	0.052
miR_135b_5p	mRNA	Znf850	-0.741	0.057
miR_133a_3p	mRNA	Serpina3f	-0.739	0.058
miR_133c	mRNA	Serpina3f	-0.739	0.058
miR_503_5p	mRNA	Esyt1	0.729	0.063
miR_133a_3p	mRNA	Ccnb1	-0.714	0.071
miR_133a_3p	mRNA	Tcap	-0.714	0.071
miR_133a_5p	mRNA	Apoa4	-0.714	0.071
miR_133a_5p	mRNA	Dpysl4	0.714	0.071
miR_133b_3p	mRNA	Golgb1	-0.714	0.071
miR_133c	mRNA	Ccnb1	-0.714	0.071
miR_133c	mRNA	Tcap	-0.714	0.071
miR_135b_5p	mRNA	Bace2	-0.714	0.071
miR_503_5p	mRNA	Dusp15	-0.714	0.071
miR_503_5p	mRNA	Tcap	0.714	0.071
miR_503_5p	mRNA	Znf850	0.704	0.077
miR_133a_5p	mRNA	Mybl2	-0.703	0.078
miR_133b_3p	mRNA	Serpina3f	-0.685	0.09
miR_133a_3p	mRNA	Znf585b	-0.679	0.094
miR_133a_5p	mRNA	Znf268	-0.679	0.094
miR_133c	mRNA	Znf585b	-0.679	0.094
miR_135b_5p	mRNA	Eln	-0.679	0.094
miR_135b_5p	mRNA	Kcnk15	0.679	0.094
miR_133a_3p	mRNA	Pi16	-0.667	0.102
miR_133a_5p	mRNA	Pi16	-0.667	0.102
miR_133c	mRNA	Pi16	-0.667	0.102
miR_503_5p	mRNA	Apoa4	0.643	0.119
miR_503_5p	mRNA	Cacna1e	0.643	0.119
miR_503_5p	mRNA	Tspan10	0.643	0.119
miR_503_5p	mRNA	Znf420	-0.643	0.119
miR_133a_3p	mRNA	Znf850	-0.63	0.129
miR_133b_3p	mRNA	Znf850	-0.63	0.129
miR_133c	mRNA	Znf850	-0.63	0.129
miR_503_5p	mRNA	Serpina3f	0.613	0.144

miR_133b_3p	mRNA	Znf585a	0.607	0.148
miR_135a_5p	mRNA	Kcnk15	0.607	0.148
miR_135a_5p	mRNA	Tcap	-0.607	0.148
miR_135b_5p	mRNA	Apoa4	-0.607	0.148
miR_135b_5p	mRNA	Cacna1e	-0.607	0.148
miR_135b_5p	mRNA	Tspan10	-0.607	0.148
miR_135b_5p	mRNA	Znf420	0.607	0.148
miR_503_5p	mRNA	Golgb1	0.607	0.148
miR_133a_3p	mRNA	Mybl2	-0.595	0.159
miR_133b_3p	mRNA	Mybl2	-0.595	0.159
miR_133c	mRNA	Mybl2	-0.595	0.159
miR_503_5p	mRNA	Mybl2	0.595	0.159
miR_133a_5p	mRNA	Esyt1	-0.591	0.162
miR_133b_3p	mRNA	Pi16	-0.577	0.175
miR_135b_5p	mRNA	Serpina3f	-0.577	0.175
miR_133a_3p	mRNA	Znf268	-0.571	0.18
miR_133b_3p	mRNA	Ccnb1	-0.571	0.18
miR_133b_3p	mRNA	Tcap	-0.571	0.18
miR_133c	mRNA	Znf268	-0.571	0.18
miR_135b_5p	mRNA	Tcap	-0.571	0.18
miR_135a_5p	mRNA	Pi16	-0.541	0.21
miR_133a_3p	mRNA	Apoa4	-0.536	0.215
miR_133a_3p	mRNA	Dpysl4	0.536	0.215
miR_133a_5p	mRNA	Kcnk15	0.536	0.215
miR_133b_3p	mRNA	Znf268	-0.536	0.215
miR_133b_3p	mRNA	Znf585b	-0.536	0.215
miR_133c	mRNA	Apoa4	-0.536	0.215
miR_133c	mRNA	Dpysl4	0.536	0.215
miR_503_5p	mRNA	Ccnb1	0.536	0.215
miR_135b_5p	mRNA	Mybl2	-0.523	0.229
miR_135a_5p	mRNA	Esyt1	-0.512	0.24
miR_133b_3p	mRNA	Apoa4	-0.5	0.253
miR_135b_5p	mRNA	Znf585a	0.5	0.253
miR_503_5p	mRNA	Znf585b	0.5	0.253
miR_133a_3p	mRNA	Esyt1	-0.493	0.261
miR_133c	mRNA	Esyt1	-0.493	0.261
miR_135b_5p	mRNA	Golgb1	-0.464	0.294
miR_135b_5p	mRNA	Dpysl4	0.429	0.337
miR_135b_5p	mRNA	Znf585b	-0.429	0.337
miR_135a_5p	mRNA	Eln	-0.393	0.383
miR_135b_5p	mRNA	Ccnb1	-0.393	0.383

miR_133b_3p	mRNA	Dpysl4	0.357	0.432
miR_135a_5p	mRNA	Bace2	-0.357	0.432
miR_133a_3p	mRNA	Kcnk15	0.321	0.482
miR_133a_5p	mRNA	Bace2	-0.321	0.482
miR_133a_5p	mRNA	Eln	-0.321	0.482
miR_133c	mRNA	Kcnk15	0.321	0.482
miR_503_5p	mRNA	Znf268	0.321	0.482
miR_135b_5p	mRNA	Esyt1	-0.315	0.491
miR_135b_5p	mRNA	Pi16	-0.306	0.504
miR_133b_3p	mRNA	Eln	-0.25	0.589
miR_133b_3p	mRNA	Kcnk15	0.25	0.589
miR_503_5p	mRNA	Pi16	0.234	0.613
miR_133a_3p	mRNA	Eln	-0.179	0.702
miR_133c	mRNA	Eln	-0.179	0.702
miR_133b_3p	mRNA	Esyt1	-0.177	0.704
miR_133a_3p	mRNA	Bace2	-0.143	0.76
miR_133c	mRNA	Bace2	-0.143	0.76
miR_135b_5p	mRNA	Znf268	-0.143	0.76
miR_133b_3p	mRNA	Bace2	-0.071	0.879

Supplementary Table S4. mRNA Sequencing Sample Quality Summary

sample	raw_reads	raw_bases	clean_reads	clean _bases	error _rate	Q20 (%)	Q30 (%)	GC_pct (%)
FRRL2300 58597-1r	89891916	13.48G	84562484	12.68G	0.03	96.95	92.06	48.53
FRRL2300 58598-1r	91288612	13.69G	88001296	13.2G	0.03	97.18	92.55	48.98
FRRL2300 58611-1r	90383924	13.56G	88614784	13.29G	0.03	96.72	91.57	45.92
FRRL2300 58595-1r	85774738	12.87G	83238604	12.49G	0.03	96.99	92.16	47.41
FRRL2300 58612-1r	86439896	12.97G	83989348	12.6G	0.03	97.31	92.94	49.03
FRRL2300 58613-1r	86503932	12.98G	84933174	12.74G	0.03	96.89	91.88	48.34
FRRL2300 58614-1r	88902398	13.34G	86910286	13.04G	0.03	97.13	92.39	49.05

Supplementary Table S5. miRNA Sequencing Sample Quality Summary

sample	reads	bases	error rate(%)	Q20(%)	Q30(%)	GC content(%)
FRRN230058597-1a	11462109	0.57G	0.01	99.32	97.52	48.93
FRRN230058598-1a	11526890	0.58G	0.01	99.32	97.28	49.08
FRRN230058611-1a	11389558	0.57G	0.01	99.41	97.77	48.94
FRRN230058595-1a	12815265	0.64G	0.01	99.33	97.56	49.2
FRRN230058612-1a	11782007	0.59G	0.01	99.32	97.48	48.98
FRRN230058613-1a	11485487	0.57G	0.01	99.41	97.75	48.81
FRRN230058614-1a	12386890	0.62G	0.01	99.41	97.69	49.23

Supplementary Table S6. mRNA Sequencing Sample Quality Verification Summary

sample	total_reads	total_map	unique_map
FRRL230058597-1r	84562484	77829195(92.04%)	72121804(85.29%)
FRRL230058598-1r	88001296	80538256(91.52%)	74105049(84.21%)
FRRL230058611-1r	88614784	79626094(89.86%)	74927967(84.55%)
FRRL230058595-1r	83238604	68540238(82.34%)	63870363(76.73%)
FRRL230058612-1r	83989348	77685877(92.49%)	71658549(85.32%)
FRRL230058613-1r	84933174	77985797(91.82%)	72465345(85.32%)
FRRL230058614-1r	86910286	80388744(92.5%)	73803183(84.92%)

Supplementary Table S7. miRNA Sequencing Sample Quality Verification Summary

sample	total sRNA	mapped sRNA	+ mapped sRNA	- mapped sRNA
FRRN230058597-1a	11111005 (100.00%)	10698883 (96.29%)	7607511 (68.47%)	3091372 (27.82%)
FRRN230058598-1a	11003576 (100.00%)	10672020 (96.99%)	7729534 (70.25%)	2942486 (26.74%)
FRRN230058611-1a	11035057 (100.00%)	10658549 (96.59%)	7578018 (68.67%)	3080531 (27.92%)
FRRN230058595-1a	12438159 (100.00%)	11417922 (91.80%)	8070588 (64.89%)	3347334 (26.91%)
FRRN230058612-1a	11377083 (100.00%)	11030633 (96.95%)	8003445 (70.35%)	3027188 (26.61%)
FRRN230058613-1a	11120620 (100.00%)	10774168 (96.88%)	7688361 (69.14%)	3085807 (27.75%)
FRRN230058614-1a	11960695 (100.00%)	11630829 (97.24%)	8239165 (68.89%)	3391664 (28.36%)

Supplementary Table S8. RT-qPCR primer sequences for *O.sibirica*.

RNAs	Forward primer	Reverse primer
ApoA4	CTAAAGCAGCCCAGTATG	CAGCACCAGTTCCAAATC
Cacna1e	TCCTGTCTCCAAGTCTACG	GTTCTCCTGTCCCTGTGA
Ccnb1	TGTGGATGAAGATGGGAAG	AGTGGAGCCAGAATGAGA
Esyt1	GCTCACTAGACACCAGAGAATT	AGTTGAAGGCTTTCCCACAT
Kcnk15	CATCTCTGCCAAGACTCA	CCTGACTTATGACCACTGA
Mybl2	TACTTCACCTGGCAAGAG	AACAGCATTCTGGCATTG
Pi16	AACCTTCATCTCCAAGTCAG	GCGTTCCCACATTCAGTA
miR-133c	ACAGCTGGTTGAAGGGGAC	GCAGGGTCCGAGGTATTC
miR-135a-5p	GCAGTCACGTATGGCTTTTT	GCAGGGTCCGAGGTATTC
miR-135b-5p	TCCCCTTCAACCAGCTG	GCAGGGTCCGAGGTATTC
miR-133b-3p	GTCCCCTTCAACCAGCTA	GCAGGGTCCGAGGTATTC
circ_0024991	TGATTGGAGGCGAGAATTGA	TGTCTCATAATGGGCAGTTGG
circ_0019806	ATCTACGAGCGCATGAATCAG	TCATTCTCATCGCCTGGTGT
circ_0007222	CTGTCTATCACCCGAGACTGCG	GGCTGGTTATTGTGCCATCTTC
